# Evaluation of antioxidant activity of nano‐ and microencapsulated rosemary (*Rosmarinus officinalis* L.) leaves extract in cress (*Lepidium sativum*) and basil (*Ocimum basilicum*) seed gums for enhancing oxidative stability of sunflower oil

**DOI:** 10.1002/fsn3.2827

**Published:** 2022-04-01

**Authors:** Seyede Zeynab Jafari, Sara Jafarian, Mohammad Hojjati, Leila Najafian

**Affiliations:** ^1^ Department of Food Science & Technology Nour Branch Islamic Azad University Nour Iran; ^2^ Department of Food Science & Technology Agricultural Sciences and Natural Resources University of Khuzestan Ahvaz Iran; ^3^ Department of Food Science & Technology Sari Branch Islamic Azad University Sari Iran

**Keywords:** antioxidant activity, *Lepidium sativum* seed gum, micro‐capsule, nano‐capsule, *Ocimum basilicum*, phenolic compounds

## Abstract

There has been interest in the use of plant extract as a natural preservative agent for improving the oxidative stability of vegetable oils. However, plant extracts have low stability against heat and environmental stress. In this study, the antioxidant potential of nano‐ and microencapsulated *Rosmarinus officinalis* L. extract (RE) obtained using the ultrasonication method was measured. The total phenolic and flavonoid content of the extract was 174.4 ± 25.9 mg gallic acid/g extract and 78.30 ± 3.2 mg rutin/g extract, respectively. Antioxidant activity of 50, 100, 200, and 400 ppm of RE was measured by DPPH free radical scavenging methods, ferric reduction assay, and β‐carotene/linoleic acid assay, and then compared to the 100 ppm of TBHQ as a common synthetic antioxidant. The results showed that the antioxidant activity increased with increasing the concentration of the extract in all evaluating methods. The antioxidant activity of 200 ppm of the free and encapsulated extract in cress (*Lepidium sativum*) and basil (*Ocimum basilicum*) seed gums at different ratios (1:0, 1:1, and 0:1) was compared to sunflower oil without antioxidants, and oil‐containing TBHQ which was stored at 60°C for 24 days. The oxidation indexes of oil samples include peroxide value, thiobarbituric acid value, and *p*‐anisidine value measured at 4‐day intervals. A lower oil oxidation was observed in oil‐containing nanoencapsulated extract followed by microencapsulated extract, free extract, and TBHQ. Since producing nanoencapsulated RE requires a higher time and speed of homogenization and due to no statistically significant difference between the antioxidant properties of nanocapsules and microcapsules in oil, the use of microcapsules of RE in basil seed gum to increase the shelf life of sunflower oil is recommended.

## Graphical abstract

Rosemary (*Rosmarinus officinalis* L.) leaves extract showed antioxidant activity. The cress (*Lepidium sativum*) and basil (*Ocimum basilicum*) seed gums are excellent coating for encapsulating rosemary extract. Oxidative stability of sunflower oil increased using encapsulated rosemary extract.

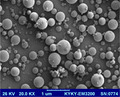

## 1 INTRODUCTION

Sunflower (*Helianthus annuus*) is one of the significant oilseeds in the world. Due to high oil yield, and lack of anti‐nutritional factors, the area under cultivation has increased (Yazdan‐Bakhsh et al., 2021). Sunflower oil (SFO) contains a high amount of linoleic acid, essential fatty acids, and vitamin E (Franco et al., 2018). It may suffer rancidity when exposed to high temperature, and the presence of oxygen which could lead to the loss of quality (Jia et al., 2021). Oil oxidation has harmful effects on human health due to the formation of rancid odors, unpleasant flavors, and discoloration (Razavi & Kenari, 2021).

However, an artificial antioxidant such as butylated hydroxytoluene (BHT), butylated hydroxyanisole (BHA), tert‐butylhydroquinone (TBHQ), and propyl gallate (PG) could inhibit oil oxidation and prolong oil shelf life (Farahmandfar et al., 2017; Jia et al., 2021). Because of the world demand for more natural antioxidants, many efforts have been devoted to finding new natural components for minimizing or replacing synthetic additives.

Rosemary (*Rosmarinus officinalis* L.) is a commonly used food‐flavoring spice plant (Moczkowska et al., 2020). It is famous as a natural antioxidant due to its strong antioxidant capacity (Yang et al., 2016). Several studies have reported the antioxidant activities of rosemary extract (Saini et al., 2020; Wang et al., 2018). It has been reported that RE could be effective in retarding oil oxidation (Chammem et al., 2015; Guo et al., 2016; Moczkowska et al., 2020; Wang et al., 2018; Yang et al., 2016). Direct use of plant extracts is not applicable because of interaction with food components, unpleasant color, odor, taste, and potential decomposition reactions during processing, and storage (Hosseinialhashemi et al., 2021). Nanoencapsulation technology providing final product functionality includes controlling the release rate, protecting food ingredients, and novel food delivery systems (Razavi et al., 2021). By using nanotechnology, extracts can be encapsulated in the form of double or multilayer nanoemulsions. Nanoemulsions are one of the most current colloidal systems for food applications. The selection of wall material as an encapsulant to fabricate nanoparticles has to be carefully made with full considerations (Razavi et al., 2020).

*Ocimum basilicum* L. (Basil) and *Lepidium sativum* L. (Cress) are commonly cultivated throughout the Middle East and Mediterranean regions. Both seeds have reasonable amounts of gums. These edible seeds are known to have health‐promoting characteristics and are used for encapsulation (Mirzabe et al., 2021).

Although the antioxidant activity of RE was investigated in several kinds of literature, there is no study on the properties of nanoencapsulated RE with BSG and CSG wall materials and its antioxidant activity on sunflower oil. Therefore, the aims of this study were to (1) determine the total phenolic content of RE, (2) investigate the antioxidant activity of RE with different methods, (3) encapsulate the RE in cress (*Lepidium sativum*) and basil (*Ocimum basilicum*) native seed gums, (4) evaluate the properties of nanoencapsulated RE, and (5) compare the antioxidant ability of TBHQ, free, and encapsulated extract of rosemary in sunflower oil during storage.

## 2 MATERIAL AND METHOD

### 2.1 Materials

The rosemary plant was obtained from Medicinal Plants Research Institute of Sari Agricultural Sciences and Natural Resources University (Sari, Iran). Basil seed gum (BSG) and cress seed gum (CSG) were obtained from Reyhan Gum Parsian Co (Tehran, Iran). Antioxidant‐free sunflower oil was obtained from the North Agro‐Industrial Oil Company. Other reagents and chemicals were purchased from Scharlau Chemie (Barcelona, Spain) or Merck Company (Darmstadt, Germany).

### 2.2 Methods

#### 2.2.1 Plant extraction and total phenolic compounds

Fifty grams of shadow‐dried rosemary was mixed with 250 ml of ethanol: water (80:20) solvent at 35°C for 30 min. The extraction was done using an ultrasonic bath (6.5l200 H, Dakshin, India) at 35 kHz of frequency. After filtering through Whatman filter paper No. 1, the solvent was evaporated using a rotary evaporator (Rotavapor R114, Waterbath B480, Büchi, Flawil, Switzerland). The final extract was kept at −18°C until further analysis (Hammi et al., 2015).

The total phenolic content of RE was determined by means of Folin–Ciocalteu reagent (Razavi & Kenari, 2021). Briefly, 50 µl of RE was added to the Folin–Ciocalteu phenol reagent (125 µl), which was diluted with distilled water in a 1:4 ratio. After 5 min, 250 µl of sodium carbonate (25%) solution was added. The solution was mixed thoroughly and then allowed to stand for 30 min at a 40°C water bath (Memmert, Schwabach, Germany). The absorbance was measured at 760 nm vs. the prepared blank which is containing all chemicals and reagents except the extract (Razavi & Kenari, 2021).

#### 2.2.2 Antioxidant activity of extract

To evaluate the antioxidant activity and optimum concentration of RE, different assessment methods were used. The DPPH radical scavenging activity and ferric reduction power were measured according to the method described by Rashidaie Abandansarie (2019). The method described by Dias et al. (2015) was used to measure the β‐carotene/linoleic acid system (Dias et al., 2015; Rashidaie Abandansarie et al., 2019). The activities were reported by the percentage of antioxidant activity. TBHQ synthetic antioxidant was applied as a positive control at 100 ppm, and concentrations of extract were 50, 100, 200, and 400 ppm. The concentration of 200 ppm of RE due to no statistically significant difference with TBHQ synthetic antioxidants was used for encapsulation of RE and addition to oil.

#### 2.2.3 Preparation of nano‐ and microcapsule of RE

The 0.05% w/v solution of BSG and CSG as coating materials at different ratios (1:0, 1:1, and 0:1) was prepared by dispersing dried powder in 40°C deionized water and after cooling mixed overnight to enhance hydration. To prepare a stable emulsion, RE (10 ml) combined with tween 80 emulsifier (40 ml) and sunflower oil (50 ml) under magnetic stirring for 15 min. Then, the nano‐ and microemulsions were created by mixing the solution in an Ultra‐Turrax homogenizer (IKA T25D, Germany) at 10,000 rpm for 5 min and 15,000 rpm for 10 min, respectively. After that, emulsions were encapsulated with 0.05% of coating materials at 1:5 ratios to form water‐in‐oil in water emulsion (Mohammadi et al., 2016; Najafi et al., 2011; Razavi & Kenari, 2021). Nano‐ and microemulsions were dried using a freeze dryer (L101, Liotop, São Carlos, Brazil) at 0.017 mPa and −57°C for 48 hr (Chranioti & Tzia, 2013).

#### 2.2.4 Particle size, zeta potential, PDI, and encapsulation efficiency of capsules

The Z‐average diameter, polydispersity index (PDI), and the zeta potential of the particles were measured using the Zetasizer (nano ZS90 equipment, Malvern, UK) based on the laser light scattering method. Samples were diluted with deionized water 10 times and placed in the tube (Joye et al., 2015). The concentration of phenolic compounds in the hexane phase was quantified, and encapsulation efficiency (EE) was calculated by Equation 1 (Kenari et al., 2020):

(1) $$EE=\frac{\mathrm{Total}\;\mathrm{phenolic}\;\mathrm{compounds}‐\mathrm{surface}\;\mathrm{phenolic}\;\mathrm{compounds}}{\mathrm{Total}\;\mathrm{phenolic}\;\mathrm{compounds}}\times 100$$

#### 2.2.5 Scanning electron microscopy (SEM)

The scanning electron microscope (S4800, Hitachi, Japan) was used to study the morphology of particles. Samples coated with a thin layer of gold and transferred to a vacuum evaporator. A beam of high‐velocity electrons with an accelerated voltage of 26 kV was applied to the samples, and the scanned images were obtained (Chatterjee & Bhattacharjee, 2013).

#### 2.2.6 Release of phenolic compounds

The particles were poured into the McCartney bottles and stored at 60 ± 2°C for 24 days, and the release properties was performed according to Esmaeilzadeh Kenari et al., (2020) at 4‐day intervals. Thus, 5 g of particles was mixed with 5 g of phosphate buffer (pH 7) and centrifuged (Centurion K 2 S series, UK) at 1500 *g* at room temperature for 90 min. Then, the lower phase was collected carefully, and total phenolic content was determined by means of Folin–Ciocalteu reagent. The amount of phenolic compounds released was determined using the following formula: Release rate (%) = 100 − (R_2_ × 100/R_1_), where R_2_ is the percentage of encapsulated compounds (total phenolics) in the outer aqueous phase, and R_1_ is the percentage of compounds in the inner phase (Kenari et al., 2020).

#### 2.2.7 Oil tests

The oil samples include (1) oil without antioxidant (CNTL), (2) oil containing 100 ppm of TBHQ (TBHQ), (3) oil containing 200 ppm of unencapsulated RE (FREE), and (4) oil containing 200 ppm of nanoencapsulated RE (NANO), (5) oil containing 200 ppm of microencapsulated RE (MICR) were placed in an incubator at a fixed temperature of 60°C for 24 days. The peroxide value (POV), thiobarbituric acid value (TBARS), and p‐anisidine value (pAnV) of samples were done according to the methods described by Chen et al. (2014) at 4‐day interval (Chen et al., 2014).

### 2.3 Statistical analysis

Statistical analysis was performed using the SPSS software Version 20.0 (Inc., Chicago, IL). The results of extract and oil samples are presented as mean ± *SD* of three replications. Comparison of the means was performed by a post‐hoc Duncan's test at 95% confidence level and 5% significance level using the one‐way ANOVA test.

## 3 RESULTS AND DISCUSSION

### 3.1 Antioxidant activity, total phenolic (TPC), and flavonoid content (TFC) of extract

The TPC and TFC of RE obtained using ultrasound were 174.25 ± 4.9 mg gallic acid/g and 78.30 ± 3.2 mg rutin/g, respectively. The previous study by Wang et al. (2018) investigated the effect of extraction time of 15, 30, 60, 120, and 150 min on the total phenolic content of RE obtained with ethanol 80%. Their results reported the amount of TPC in the range of 10.30–160.70 mg GA/g extract, which is lower than the amount reported in the current study (Wang et al., 2018). Similarly Saini et al. (2020) reported TPC and TFC for rosemary leave extract to be 136.66 ± 7.41 mg gallic acid/g and 37.13 ± 6.04 mg rutin/g, respectively (Saini et al., 2020). The TPC of rosemary extract reported by other researcher were 32.42 mg GA/gDM (Moczkowska et al., 2020), 112.0 mg GAE/g (Chammem et al., 2015), and 162.0 mg GAE/g extract (Erkan et al., 2008). The TFC of rosemary extract was reported 24 mg CE/g extract by Wang et al. (2018) (Wang et al., 2018). The reason for the difference may be related to the growth conditions, harvest season, plant variety, genetics, extraction conditions, polarity, and type of solvent (Erkan et al., 2008; Yazdan‐Bakhsh et al., 2021).

**FIGURE 1:**
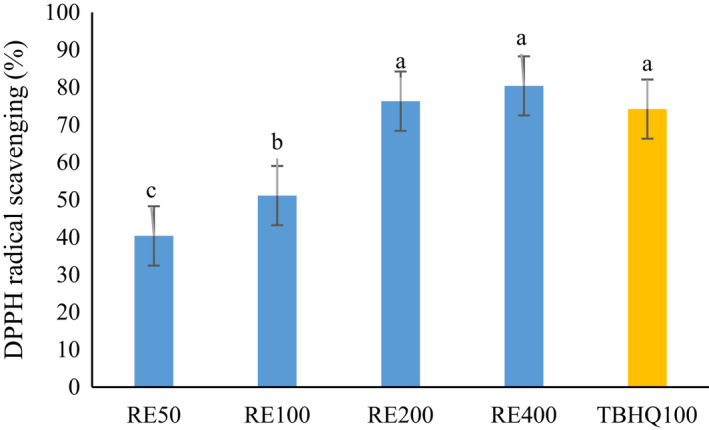
DPPH radical scavenging of *Rosmarinus officinalis* L. leave extract. Different letters indicate significant statistical difference between samples at *p* < .05

**FIGURE 2:**
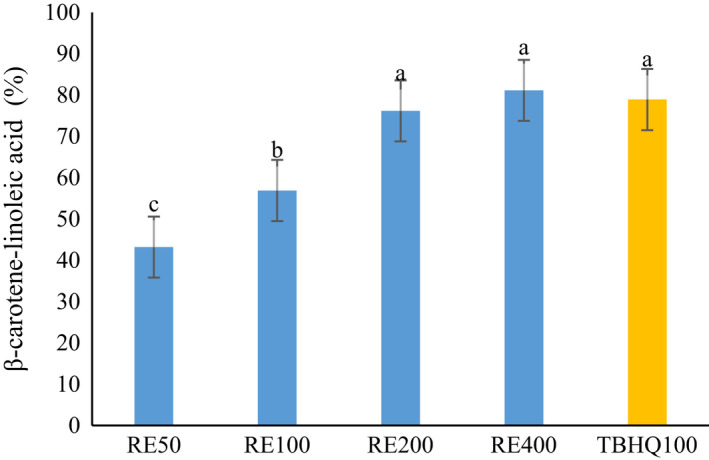
β‐carotene/linoleic acid bleaching activity of *Rosmarinus officinalis* L. leave extract. Different letters indicate significant statistical difference between samples at *p* < .05

**FIGURE 3:**
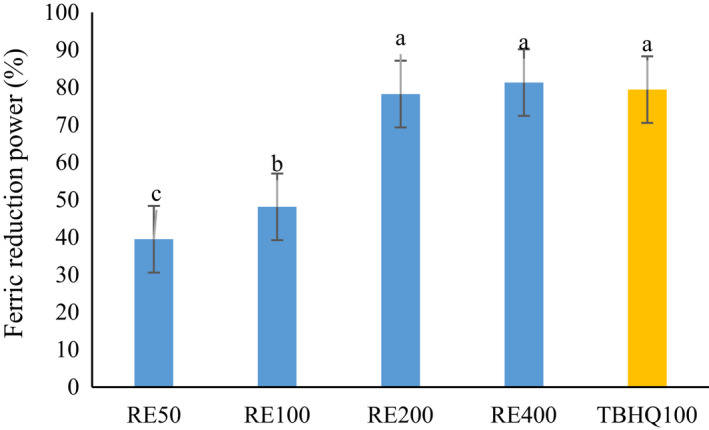
Ferric reduction antioxidant power of *Rosmarinus officinalis* L. leave extract. Different letters indicate significant statistical difference between samples at *p* < .05

Antioxidant activity of RE was evaluated by the DPPH radical scavenging activity, β‐carotene/linoleic acid assay, and ferric reduction power. As can be seen in Figures 1, 2, and 3, by increasing the concentration of extract from 50 to 400 mg/L, the antioxidant activity in all methods was increased. No statistically significant difference was observed between TBHQ and RE at 200 and 400 mg/L of concentration. Chammem et al. (2015) investigated the radical scavenging activity of different concentrations (0.02%, 0.05%, and 0.08%) of rosemary extract comparatively to a synthetic BHT antioxidant. A relatively high antioxidant activity is noticed for rosemary extract (Chammem et al., 2015). There is a relationship between the concentration of phenolic compounds of extract and the antioxidant activity of phenolic compounds depends on their structure (Esmaeilzadeh Kenari & Razavi, 2022; Farahmandfar et al., 2019; Razavi & Kenari, 2021). RE contains compounds which are responsible for a free radical capture and therefore could be used to stabilize and retard oil oxidation.

The RE has ability to reduce Fe^3+^ to Fe^2+^. Moczkowska et al. (2020) reported higher degree of Fe^3+^ reduction at higher concentration of rosemary extract (Moczkowska et al., 2020). Pires et al. (2017) indicated that RE showed lower values from the FRAP assay compared with BHA synthetic antioxidant and even green tea extract. They stated that the antioxidant activity of different extracts was directly related to the amounts of active components, which, these active compounds in rosemary extract were much lower than green tea extract (Pires et al., 2017). Rashidaie Abandansarie et al. (2019) reported a rising trend in antioxidant activity of RE by increasing the concentration from 100 to 1600 ppm and also higher antioxidant activity than TBHQ in both FRAP and DPPH radical scavenging method (Rashidaie Abandansarie et al., 2019). The results of β‐carotene/linoleic acid assay showed that the RE has ability in preventing lipid peroxidation of linoleic acid by averting the discoloration of β‐carotene (Dias et al., 2015).

These results are consistent with the results of Saini et al. (2020), who reported that increasing the concentration of the RE significantly increase the radical scavenging activity and ferric reducing antioxidant power. In addition, the RE has capable of neutralizing the DPPH free radicals via hydrogen donating ability. They also reported an increase in absorbance of RE with increase in concentration from 20 to 100 mg/L in FRAP assay (Saini et al., 2020). The main antioxidant that is present in rosemary is carnosic acid which is responsible for over 90% of the antioxidant properties of rosemary extract (Chen et al., 2014; Moczkowska et al., 2020).

### 3.2 Characteristics of nanocapsules and encapsulation efficiency

Determination of particle size, PDI, zeta potential, and encapsulation efficiency of particles are very important factors which affect the stability of colloidal system and properties of final product. The results of particle size, PDI, zeta potential, and encapsulation efficiency of encapsulated RE are shown in Table 1. All nanocapsules and microcapsules have nanometric (284.2–317.3 nm) and micrometric diameters (1002.5–1236.2 µm), respectively. Rashidaie Abandansarie et al. (2019) reported the diameter of RE in basil seed gum wall 154.69 nm which is smaller than diameter of nanocapsules prepared in the current study (Rashidaie Abandansarie et al., 2019). The type of ingredients is an effective parameter on particle diameter of capsules.

**TABLE 1:** Z‐average size, PDI, zeta potential, and encapsulation efficiency of different particles

Sample	Z‐average size (nm)	PDI	Zeta potential (mv)	Encapsulation efficiency (%)
NCSG	317.3^a^	0.284^c^	−31.19^a^	50.25^c^
NBSG	284.2^c^	0.300^a^	−35.24^c^	56.36^a^
NMIX	301.4^b^	0.298^b^	−34.77^b^	54.88^b^
MCSG	1236.2^A^	0.229^C^	−31.01^A^	55.42^C^
MBSG	1002.5^C^	0.247^A^	−35.72^C^	61.17^A^
MMIX	1111.7^B^	0.238^B^	−34.96^B^	59.25^B^

Note

Different lower case letters indicate the significant statistical differences at *p* < .05 between nanocapsules. Different upper case letters indicate the significant statistical differences at *p* < .05 between microcapsules.

PDI shows the dispersity of particles. The PDI values vary from 0.0 to 1.0. The PDI of different particles ranged from 0.229 to 0.300. A higher PDI was observed in particle prepared with basil seed gum. Also, nanocapsules exhibited higher PDI. All particles had the PDI index ≤0.300, which shows monotone distribution. An increase in particle diameter caused a decrease in PDI. These results are in agreement with a study reported by Chaari et al. (2018). They reported lower PDI in microemulsions of carotenoids in comparison with nanoemulsions and also slight decrease in PDI of microemulsions by increasing the particle diameter (Chaari et al., 2018). A study stated that the encapsulated extract in particle with shahi (*Lepidium sativum*) seed gum wall had the largest particle size than that of encapsulated (Yazdan‐Bakhsh et al., 2021).

The zeta potential of particles varied from −35.24 to −31.19 mv in nanocapsules and from −35.72 to −31.01 mv in microparticles. The negative zeta potential of all particles is related to the presence of anionic groups in seed gum (Taheri & Jafari, 2019) and RE. The results of encapsulation efficiency of all samples showed higher encapsulation efficiency (50.0% ≤) which indicated the suitable encapsulating conditions. Other literatures also reported higher than 50% of encapsulation efficiency for particles with polysaccharide wall materials (Rashidaie Abandansarie et al., 2019; Razavi & Kenari, 2021). Higher viscosity of wall material caused an increase in encapsulation efficiency of particles (Razavi et al., 2021). Therefore, the particles prepared by basil seed gum showed a higher encapsulation efficiency of extract.

### 3.3 Morphology of particles

**FIGURE 4:**
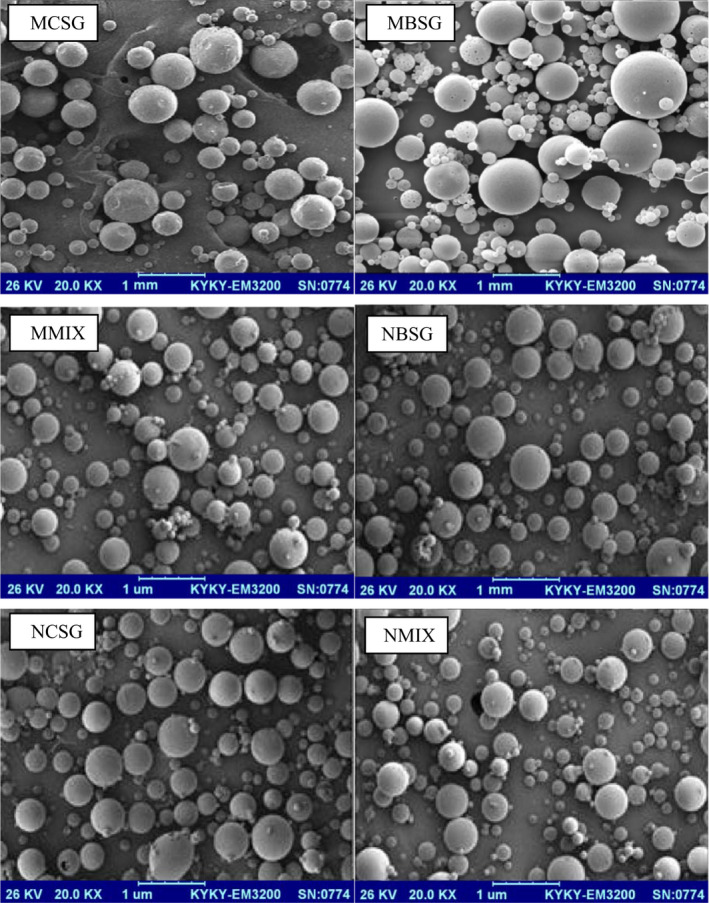
SEM images of encapsulated *Rosmarinus officinalis* L. leave extract in different wall materials: MCSG, microcapsule of RE in cress seed gum; MBSG, microcapsule of RE in basil seed gum; and MMIX, microcapsule of RE in basil seed gum/cress seed gum; NCSG, nanocapsule of RE in cress seed gum; NBSG, nanocapsule of RE in basil seed gum; and NMIX, nanocapsule of RE in basil seed gum/cress seed gum

A microstructural visualization was performed to study the surface morphology of the cress/basil seed gum particles containing RE. The results of scanning electron microscopy *(SEM*) images of samples are illustrated in Figure 4. All particles were spherical with a smooth surface. No significant differences between particles were observed. This result is in accordance with the previously published literature explaining that particles produced using emulsification method are often spherical (Kenari et al., 2020; Razavi & Kenari, 2021; Yazdan‐Bakhsh et al., 2021).

### 3.4 Release rate

Determination of phenolic compounds release rate of particles during storage time is very important. The controlled release of phenolic compounds during incubation period affects the antioxidant activity of particles and shelf life of products. The results of phenolic compounds release from different particles during incubation period are shown in Table 2. As can be seen, in all particles, phenolic compounds were released over time and statistical differences (*p* < .05) were observed. The size of particles is critical factor which affects the release rate of phenolic compounds and small particles exhibited higher amount of phenolic content. The samples of NBSG and MBSG due to lower rate of release at the end of incubation period were selected for addition in sunflower oil.

**TABLE 2:** The release of phenolic compounds from encapsulated rosemary extract during storage (%)

Sample	0	4	8	12	16	20	24
NCSG	2.12 Ga	4.16^Fa^	7.88^Ea^	12.16 Da	17.21^Ca^	20.88^Ba^	24.16^Ab^
NBSG	2.01 Ga	3.52^Fbc^	5.16^Ec^	9.48^Dc^	13.88^Cc^	16.65^Bd^	20.72^Ae^
NMIX	2.05 Ga	3.81^Fab^	6.46^Ebc^	10.73^Dbc^	15.43^Cb^	18.73^Bb^	22.41^Ac^
MCSG	2.16 Ga	3.17^Fcd^	4.84^Ecd^	8.53^Dd^	13.12^Cc^	17.43^Bc^	22.89^Abc^
MBSG	2.15 Ga	3.09^Fd^	4.59^Ed^	8.06^Dd^	10.68^Ce^	14.36^Bf^	20.43^Ab^
MMIX	2.19 Ga	3.12^Fcd^	4.63^Ed^	8.37^Dd^	11.11^Cd^	15.09^Be^	21.56^Ad^

Note

Different lower case letters indicate the significant statistical differences at *p* < .05 between samples. Different upper case letters indicate the significant statistical differences at *p* < .05 during incubation period.

### 3.5 Oil tests

#### 3.5.1 Peroxide value

**FIGURE 5:**
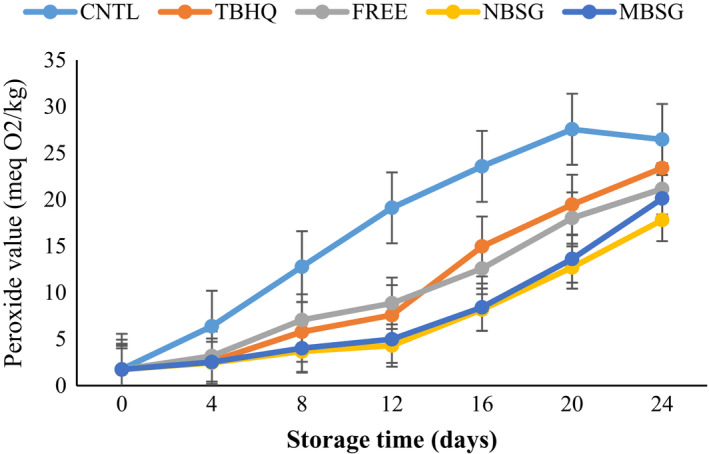
Change in peroxide value of different SFO samples during incubation, CNTL = oil without antioxidant, TBHQ = oil containing 100 ppm of TBHQ, FREE = oil containing 200 ppm of free RE extract, NBSG = oil containing 200 ppm of NBSG nanocapsules, MSG = oil containing 200 ppm of NBSG microcapsules

Peroxide value (POV) determines the concentration of peroxides in the early stages of lipid oxidation. A lower POV implies a higher oxidative stability (Ganji & Sayyed‐Alangi, 2017; Yang et al., 2016). The changes in POV of different oil samples during incubation period are shown in Figure 5. It can be seen that in all sunflower oil samples, the peroxide value has increased over time (Asadi & Farahmandfar, 2020; Chammem et al., 2015; Farahmandfar & Ramezanizadeh, 2018) and the significant statistical difference was observed at different incubation periods except days 0 and 4 of oil samples containing antioxidant. The control sample had the highest POV. Similarly, Yang et al. (2016) reported that during 24‐day storage, POVs of the soybean oil samples with and without added antioxidants increased sharply and oil with rosemary extract showed lower oxidation (Yang et al., 2016). The drop in POV of CNTL sample at the end of storage period was due to the unstable primary oxidation products that are sensitive to decomposition. These products form the carbonyl compounds (Yang et al., 2016). In all incubation periods, the blank sunflower oil had significantly (*p* < .05) higher POVs than oil containing antioxidants. The lower POV of oil with plant extract than oil with synthetic antioxidant during frying process or storage also reported by other researchers (Agregán et al., 2017; Farahmandfar et al., 2015; Razavi & Kenari, 2021; Yazdan‐Bakhsh et al., 2021). As can be seen in Figure 5, encapsulated rosemary exhibited higher antioxidant activity than synthetic antioxidant which is in line with the findings in a recent study by other researchers for *Mentha piperita* (Royshanpour et al., 2021) extract and nanoencapsulated *Ferula persica* extract (Estakhr et al., 2020).

#### 3.5.2 Thiobarbituric acid value

**FIGURE 6:**
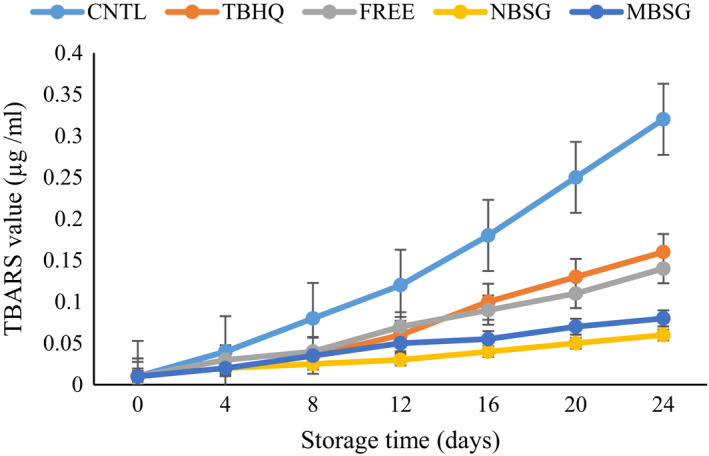
Change in thiobarbituric acid value of different SFO samples during incubation, CNTL = oil without antioxidant, TBHQ = oil containing 100 ppm of TBHQ, FREE = oil containing 200 ppm of free RE extract, NBSG = oil containing 200 ppm of NBSG nanocapsules, MSG = oil containing 200 ppm of NBSG microcapsules

The content of secondary products of oxidation like malondialdehyde which leads to the formation of off‐flavors in oil was expressed by the thiobarbituric acid reactive substance—TBARS (Moczkowska et al., 2020). The results of change in TBARS of oil samples during incubation period are shown in Figure 6. A difference in the TBARS between day 0 and day 24 of all samples was noted. Oil sample without antioxidant had a significantly higher TBARS. These results are in agreement with Moczkowska et al. (2020), who revealed a lower TBARS in palm oil containing rosemary extract in comparison with oil without antioxidant or BHT (Moczkowska et al., 2020). The samples with an addition of encapsulated RE had a decreased TBARS compared to the CNTL, TBHQ, and FREE samples. Similarity, Chen et al. (2014) investigated the effect of RE, BHA, BHT, and TBHQ on TBARS of sunflower oil at 60 ºC for 24 days. Their results showed that TBARS gradually increased in sunflower oil during storage period and increased acceleration after the 4th day. They also reported maximum TBARS for control sample (Chen et al., 2014), which is in line with the results of current study. Previous studies by other researchers also showed that encapsulated extract of *Heracleum lasiopetalum* (Yazdan‐Bakhsh et al., 2021) and *Fumaria parviflora* (Razavi & Kenari, 2021) can retard oil oxidation in terms of TBARS and extend the shelf life of sunflower oil.

#### 3.5.3 P*‐*anisidine value

**FIGURE 7:**
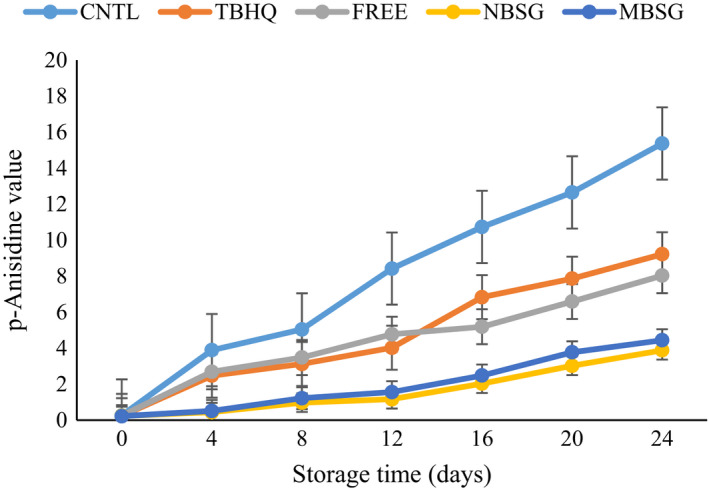
Change in *p*‐anisidine value of different SFO samples during incubation, CNTL = oil without antioxidant, TBHQ = oil containing 100 ppm of TBHQ, FREE = oil containing 200 ppm of free RE extract, NBSG = oil containing 200 ppm of NBSG nanocapsules, MSG = oil containing 200 ppm of NBSG microcapsules

P‐anisidine analysis is suitable method to evaluate the secondary lipid oxidation. As oil oxidation continues, the formed peroxides were no longer assayed. The color changes which occurred due to aldehyde‐carbonyl bound generation during secondary lipid oxidation were measured as a P‐anisidine value (Guo et al., 2016). The results of changes in the P‐anisidine value (PAnV) of different oil samples during incubation period are illustrated in Figure 7. In this study, as expected, there was a statistically significant (*p* < .05) increase in PAnV throughout the incubation period, regardless of samples treated with antioxidant. CNTL oil showed the highest PAnV, which is in line with the result reported by Moczkowska et al., (2020). A higher PAnV indicates that higher rancid oil is produced. Guo et al. (2016) revealed that the highest inhibitory effect on the generation of secondary oxidation products was observed in the palm oil containing natural antioxidant rosemary extract (Guo et al., 2016). Dias et al. (2015) studied the antioxidant activity of rosemary extract in soybean oil under accelerated heating, compared with synthetic antioxidant TBHQ. The results in this study were similar to theirs, and RE was more effective than TBHQ in decreasing the PAnV of oil (Dias et al., 2015). In a study by specified that during incubation, flaxseed oil with an addition of RE had the lowest POV and PAnV which is related to phenolic compounds specially carnosol and rosmarinic acid (Chen et al., 2014; Wang et al., 2018). Unsurprisingly, oil without antioxidant was the most easily oxidized sample. The samples of POV, TBARS, and PAnV with RE incorporated were significantly lower than that of oil with added TBHQ synthetic antioxidant, indicating that RE was more effective in stabilizing oil against oxidative deterioration compared to synthetic antioxidant. The antioxidant activity of TBHQ is because of its pure phenolic compound. However, RE is a sort of crude extract and it contained a large amount of the mixture of phenolic compounds with synergy effect (Guo et al., 2016). The antioxidant activity of phenols is due to their scavenging ability on free radicals, chelate metals, and donate electron or hydrogen atoms (Chen et al., 2014).

## 4 CONCLUSION

The effect of different wall materials on the characteristics of nano‐ and microcapsules of RE and the effect of free and encapsulated RE on the stability of sunflower oil during incubation period were investigated. The highest antioxidant properties were noticed for nanoencapsulated extract followed by microencapsulated extract and the lowest for TBHQ. The addition of antioxidants, whether TBHQ or RE, had a significant effect on the stability of sunflower oil during incubation period. From the results obtained in the current study, RE had the greatest antioxidant activity in inhibiting the oxidation process of sunflower oil, but RE in both nano‐ and microcapsules forms had the highest antioxidant properties. It is related to the controlled release of phenolic compounds from particles during incubation period. Considering that the production of nanocapsules requires higher homogenization time and speed, but their antioxidant impacts in sunflower oil were not statistically significant. Therefore, the results of current study suggest the use of microencapsulated RE in basil (*Ocimum basilicum*) seed gum for increasing shelf life of sunflower oil instead of synthetic TBHQ antioxidant.

## AUTHOR CONTRIBUTION

**Seyede Zeynab Jafari:** Conceptualization (equal); Investigation (equal). **Sara Jafarian:** Data curation (equal); Supervision (equal); Visualization (equal); Writing – review & editing (equal). **Mohammad Hojjati:** Methodology (equal); Supervision (equal); Writing – original draft (lead); Writing – review & editing (equal). **Leila Najafian:** Formal analysis (equal); Methodology (equal).
